# Surgical treatment of internal hernia after Roux–en-Y gastric bypass — impact of institutional standards and surgical approach

**DOI:** 10.1007/s00423-023-03049-2

**Published:** 2023-08-17

**Authors:** Lars Kollmann, Johan F. Lock, Cathérine Kollmann, Miljana Vladimirov, Christoph-Thomas Germer, Florian Seyfried

**Affiliations:** 1 Department of General, Visceral, Transplantation, Vascular and Pediatric Surgery, Center of Operative Medicine (ZOM), University Hospital of Würzburg, Würzburg, Germany; 2 Department of General, Visceral and Thoracic Surgery, Hospital of Nuernberg, Nuernberg, Germany

**Keywords:** Internal hernia, Gastric bypass, Bariatric surgery, Standardization, Quality improvement

## Abstract

**Introduction:**

Internal hernia is one of the most frequent long-term complications after laparoscopic gastric bypass surgery (RYGB). Surgical treatment of an internal hernia itself has risks that can largely be avoided by the implementation of institutional standards and a structured approach.

**Material and methods:**

From 2012 until 2022, we extracted all consecutive bariatric cases from the prospectively collected national database (StuDoQ). Data from all patients undergoing internal hernia repair were then collected from our hospital information management system and retrospectively analyzed. We compared patient characteristics and surgical outcome of patients before and after the implementation of standard operating procedures for institutional and perioperative aspects (first vs. second time span).

**Results:**

Overall, 37 patients were identified (median age 43 years, 86.5% female). Internal hernia was diagnosed after substantial weight loss (17.2 kg/m^2^) and on average about 34 months after RYGB. Baseline characteristics (age, sex, BMI, achieved total weight loss% and time interval to index surgery were comparable between the two groups). After local standardization, the conversion rate decreased from 52.6 to 5.6% (*p* = 0.007); duration of surgery from 92 to 39 min (*p* = 0.003), and length of stay from 7.7 to 2.8 days (*p* = 0.019).

**Conclusion:**

In this study, we could demonstrate that the surgical therapy of internal hernia after gastric bypass can be significantly improved by implementing institutional and surgical standards. The details described (including a video) may provide valuable information for non-specialized surgeons to avoid pitfalls and improve surgical outcomes.

**Supplementary Information:**

The online version contains supplementary material available at 10.1007/s00423-023-03049-2.

## Introduction

Bariatric surgery is currently the most effective treatment for severe obesity and its related co-morbidities [1]. As a result, the number of bariatric procedures being performed worldwide is constantly rising [2]. Despite its clear health benefits, the life-time risk of undergoing additional abdominal surgery after bariatric surgery is doubled [3]. The reasons for this vary widely, including additional abdominal surgery including cholecystectomy for symptomatic gall stones, revisional procedures due to reflux and insufficient treatment of obesity or its associated comorbidities [4]. While immediate complications requiring revisional surgery after bariatric surgery are rare, long-term complications are more frequent [5].

The most common long-term complication after Roux-en-Y gastric bypass is an internal hernia, which usually occurs metachronously after relevant weight loss [6]. Numerous prospective randomized studies have shown an advantage for the primary closure of the mesenterial defect during the initial procedure [7, 8]. However, the closure itself contains some morbidity, and an internal hernia can still occur. The incidence of an internal hernia without mesenterial defect closure is up to 16% of cases [9].

The typical symptoms of hernia include moderate to severe intermittent abdominal pain in the left upper quadrant along with vomiting [10]. However, symptoms can be variable and in the case of an ileus or incarceration, the patient might present with an acute abdomen [10]. If clinically suspected, computed tomography of the abdomen is recommended as its sensitivity, and specificity has been reported to exceed 80% [11]. In unclear cases, however, a diagnostic laparoscopy should immediately be performed.

The surgical treatment of an internal hernia itself carries the risk for relevant morbidity [12]. Certain pitfalls can largely be avoided by the implementation of institutional standards and a stringent and targeted approach [13]. At certified centers for bariatric surgery, cases are handled more efficiently and with lower morbidity due to the availability of experienced surgeons [12, 13]. However, not all of these emergency cases are treated in certified centers. Thus, non-specialized or less experienced surgeons may also be increasingly confronted with long-term complications and emergencies resulting from bariatric surgery.

During the past 10 years, we have modified some institutional and perioperative standards for patients diagnosed with internal hernia after Roux-en-Y gastric bypass. For quality assurance, we aimed to investigate their influence on the quality of patient care. Further, we systematically present the perioperative setup and a novel step-by-step approach to perform surgery safely.

## Material and methods

From January 2012 until July 2017, surgery was performed by the on-call surgeon in a non-standardized and non-targeted laparoscopic approach. The primary aim was to identify the internal hernia before closing the mesenteric defect with non-absorbable running suture. Since August 2017, we have standardized some key institutional and perioperative standards for patients diagnosed with internal hernia after Roux-en-Y gastric bypass:

- All revisional surgeries for suspected IH were performed by a certified senior bariatric surgeon.
- For patient placement and surgery, standardized operating procedures were implemented with the primary aim to safely replace the small intestine beginning at the terminal ileum, before investigating the alimentary, biliopancreatic limb, and the mesenterial gaps.

### Patient positioning

**Fig. 1:**
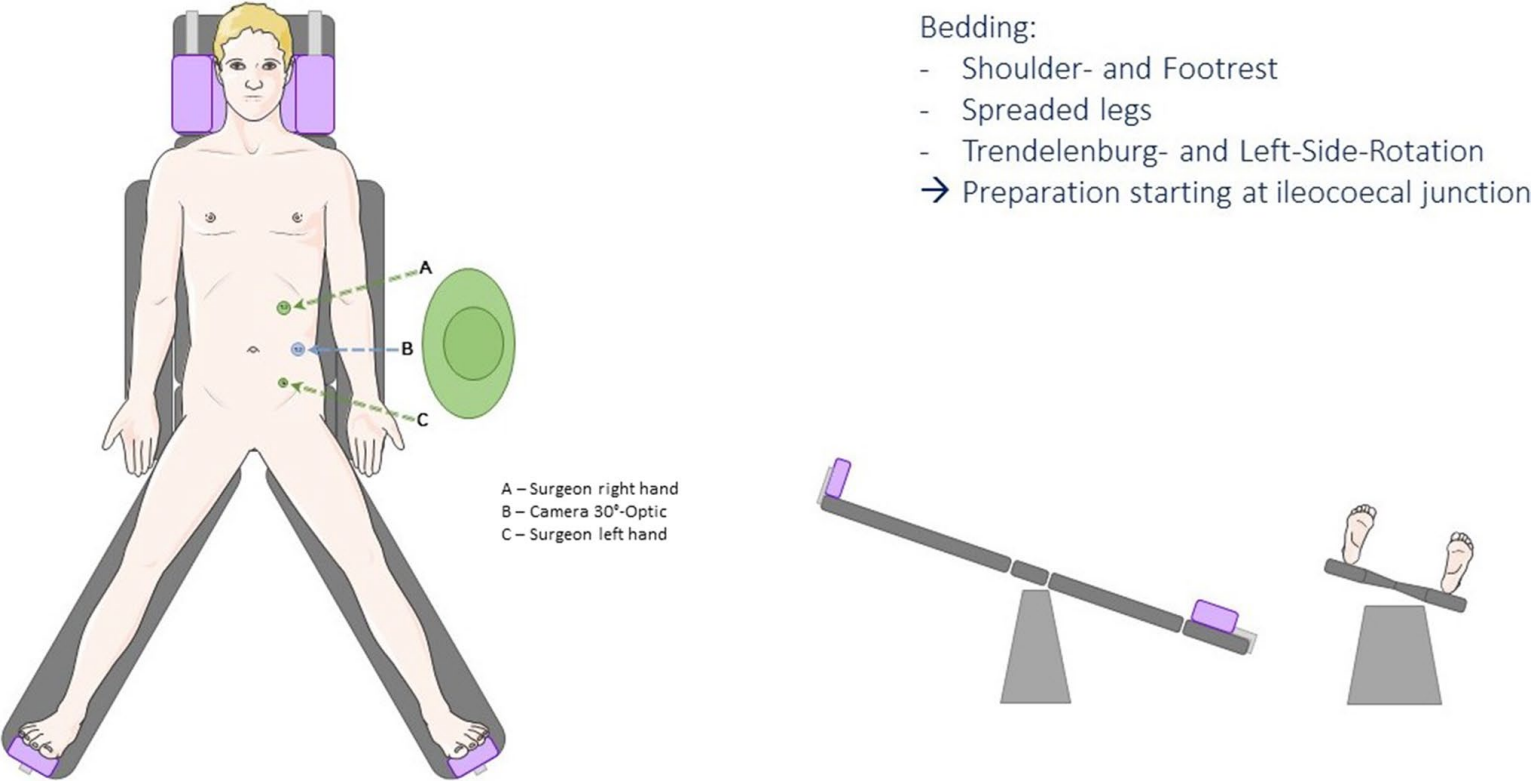
Exploration of the common channel

Changing patient position during surgery from a regular supine over a Trendelenburg left-sided to a split leg reverse Trendelenburg’s position (Fig. 1 a and b) was found to be beneficial. Therefore, footrests as well as a padded head, and neckrest were used. To prevent arm plexus lesions, the patient’s arms were tucked by their sides with bedsheets (Fig. 1a).

### Team position and port placement

**Fig. 2:**
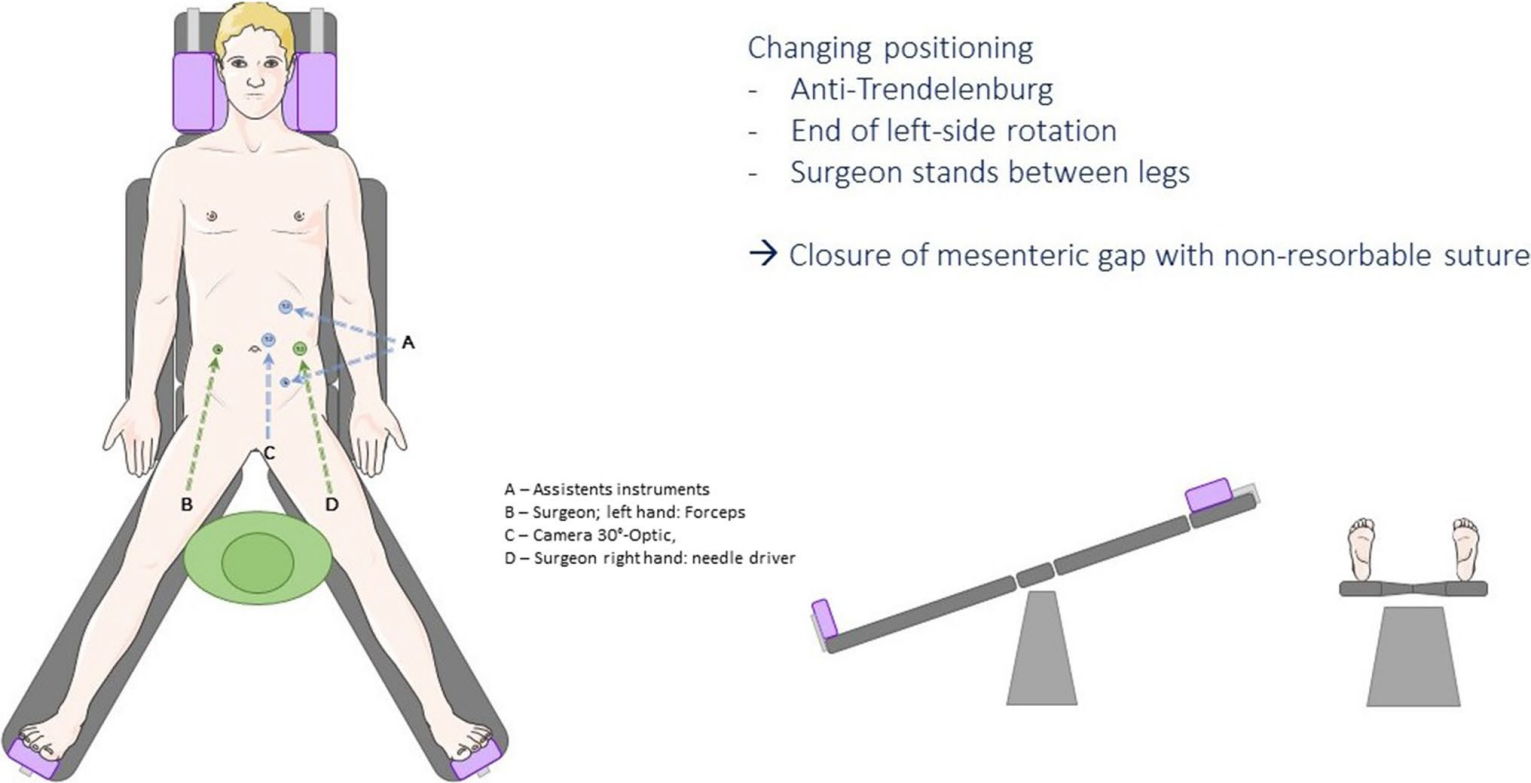
Exploration of the alimentary and biliopancreatic limb, closure of the mesenteric gap

The surgeon and the camera-assistant started surgery at the left side of the patient. The first port (12mmXCel) was placed in the left upper quadrant (Palmar’s point), and the capnoperitoneum was established. The second port (12 mm XCel) was inserted into the left lateral abdomen on umbilical level and the third port (5 mm) into the left lower quadrant (Fig. 2a). The camera was then placed into the second trocar.

### Exploration of the common channel

The patient was next positioned in a Trendelenburg (15°) left-sided position, and the terminal ileum was identified. The common channel starting from the terminal ileum was then constantly and carefully followed while it was gently replaced in the right lower quadrant (Video) until the jejuno-jejunostomy was reached.

### Exploration of the alimentary limb, the biliopancreatic limb, and the mesenteric gaps

When the jejuno-jejunostomy was reached, two more ports were placed as shown in Fig. 2b. Then, the patient’s position was changed to a reverse Trendelenburg’s position. The surgeon then stood between the split legs with the assistant to the right side (Figs. 1b and 2b). The camera position also changed according to Fig. 2b.

The view was then optimized for exploration of the alimentary limb, the biliopancreatic limb and the mesenteric gaps. The alimentary limb was identified and followed starting from the pouch until the jejuno-jejunostomy was reached. The potential mesenteric gap at the jejuno-jejunostomy was then explored. The use of atraumatic holding instruments over ports 1 and 2 (Fig. 2b) ensured easy exposure and closure of the mesenteric defect. Both mesenteric gaps were closed by non-resorbable running sutures (Ethibond 2–0) beginning at the intestine. At this site of the mesenteric gap, a small gap was left open (about 5–10 mm) to prevent bowel obstructions due to kinking.

Before closing the Petersen’s space, it was assured that the biliopancreatic channel was correctly placed at the right side on the video screen which is the patients’ left side of the mesenteric defect (Video).

For sufficient closure, the gap between the transverse mesocolon and the mesenterium of the alimentary limb was closed. The gap between the remnant stomach and alimentary limb remained open as it is in our experience a very uncommon cause for an internal hernia.

### Data assembly and statistical analyses

This retrospective single-center study covered the period from January 2012 until October 2022 in our tertiary center (University Hospital Würzburg, Germany). Since 2014, our clinic is a certified center of reference for metabolic and bariatric surgery by the German society of general and visceral surgery (DGAV).

From January 2012 until October 2022, we extracted all consecutive bariatric cases from the prospectively collected national database (StuDoQ). Of those (*n* = 1143), we identified 37 patients who received surgery for internal hernia after Roux-en-Y gastric bypass. Data on patient characteristics, clinical presentation, diagnostic results, and operative outcome after internal hernia repair were then collected from our hospital information management system and retrospectively analyzed.

We compared patient characteristics and surgical outcome of patients before and after the implementation of standard operating procedures for institutional and perioperative aspects for patients diagnosed with internal hernia after Roux-en-Y gastric bypass (first vs. second time span).

All statistical analyses were performed using IBM SPSS Statistics 28 (International Business Machines Corporation, Armonk, NY). Descriptive data are reported as means with standard deviations, unless otherwise stated. Comparisons between the analyzed cohorts were performed using chi-square, Fisher’s exact, Mann–Whitney *U* tests or one-way analysis of variance, in accordance with data scale and distribution. The time-intervals were compared by Kaplan–Meier analysis with log rank test. The level of statistical significance was 0.05 (two-sided).

## Results

### Patient characteristics

Patient characteristics are shown in detail in Table 1. Median age was 43 years (range 24–65) with no differences between the two different groups (*p* = 0.54). The majority of patients were female 32 (86.5%) with no differences between groups (*p* = 0.68). Body mass index (BMI) at baseline before Roux-en-Y gastric bypass was 49.3 kg/m^2^ and tended to be higher in the first time span group (51.7 kg/m^2^ vs. 46.8 kg/m^2^, *p* = 0.052). Weight loss was 17.2 kg/m^2^ and not different between the groups (p = 0.52). Accordingly, BMI at surgery for internal hernia was 32.9 kg/m^2^ overall and not different between groups (*p* = 0.20). Time interval from index surgery to internal hernia repair was 34 (0–124) months and not different between the groups (*p* = 0.43). All other patient characteristics (comorbidities, etc.) did not differ between groups (data not shown).

**Table 1:** Description of patients’ characteristics, clinical presentation and surgical outcome before/after standardization

Patient characteristics	Total [*n* = 37]	2012–2017 [*n* = 19]	2017–2022 [*n* = 18]	*p* value
Age (median)	43 (24–65)	44 (30–65)	42 (24–54)	0.54
Sex m/f (%)	5/32 (13.5 /86.5)	3/16 (16/84)	2 /16 (11/89)	0.68
BMI at index RYGB (kg/m^2^)	49.3 (32.0–69.1)	51.7 (40.1–69.1)	46.8 (32.0–61.6)	0.052
BMI at internal hernia revision (kg/m^2^)	32.9 (20.4–54.7)	34.5 (21.8–54.7)	31.2 (20.4–45))	0.20
Weight loss since index surgery (BMI points)	17.2 (+ 2.7–33.8)	17.1 (0–30)	17.2 (+ 2.7–33.8)	0.96
Time interval from RYGB (months)	34 (0–124)	30 (0–94)	38 (6–124)	0.43
Clinical appearance				
Abdominal pain	37 (100)	19 (100)	18 (100)	1
Intermittent abdominal pain	18/37 (48.6)	7/19 (36.8)	11/18 (61.1)	0.13
CT scan result: hurricane-eye-sign	31/36 (86.1)	16/19 (84.2)	15/17 (88.2)	0.55
Free-abdominal-fluid	18/36 (50)	10/19 (52.6)	8/17 (47.1))	0.55
SWIRL score > 1	24/37 (64.9)	13/19 (68.4)	11/17 (61.1)	0.68
SWIRL score > 2	8/37 (21.6)	2/19 (10.5)	6/17 (33.3)	**0.042**
Diameter intestine in mm (CT-scan)	26 (16–55)	28 (16–55)	25 (19–44)	0.49
Emergency surgery (< 6 h)	31 (83.8)	16 (84.2)	15 (83.3)	0.94
Operative outcome				
Conversion or primary open^1^	11 (29.7)	10 (52.6)	1 (5.6)	**0.007**
Duration of surgery (min)	53 (30–134)	92 (41–134)	39 (30–117)	**0.003**
Clavien-Dindo classification				
< Grade IIIa (%)	4 (10.8)	4 (21.1)	0	**0.012**
Grade IIIb (%)	1 (2.7)	1 (5.3)	0	0.85
> Grade IIIb (%)	0	0	0	1
CCI (MW 95% CI)	3,5 (0–33.7)	6.4 (0–33.7)	0.5 (0–8.7)	0.065
Length-of-stay, days	5.3 (1–35)	7.7 (2–35)	2.8 (1–12)	**0.019**
In-hospital mortality	0	0	0	1

^1^One case primary open surgery

### Clinical presentation and diagnostic results

All patients were symptomatic and presented with abdominal pain. Intermittent pain was reported by 18 patients (48%) with no significant differences between groups (*p* = 0.13). All but one patient received an abdominal computed tomography (CT) scan. Free abdominal fluid was detected in 18 patients (50%), with no differences between groups (*p* = 0.55). Hurricane-eye-sign was detected in 31 patients (86.1%) which did not differ between groups (*p* = 0.547). Median diameter of the intestine was 26 mm (16–55) overall and not different between groups (*p* = 0.487).

The SWIRL score, a radiologic scoring system predicting internal hernia with CT-scan results and clinical appearance [14], was 2 (median) with no differences between groups. SWIRL Score > 1 was reached in 24 patients (64.9%), with no differences between groups (*p* = 0.679).

SWIRL Score > 2 was present in 8 patients (21.6%) overall, with two (10.5%) in the first and six (33.3%) in the second group (*p* = 0.042).

### Operative outcome

Overall, 31 patients (83.8%) underwent emergency surgery (< 6 h), with no differences between groups (*p* = 0.94). All but one patient received a laparoscopic approach due to multiple previous open surgeries.

Conversion to open surgery was required in 29.7% (11/37), with 52.6% (10/19) in the first and 5.6% (1/18) in the second timespan (*p* = 0.007). A detailed description of all cases requiring conversion to open surgery is provided in Table 2. Lesions of the small intestine were the cause in 5 out of 11 patients. Reduced mobility of the small intestine was listed as the cause in 4/11 cases; fragility of the intestine and fear of lesioning were the reason for conversion in 3/11 times. For the large part, a combination of different causes lead to conversion. Overall, duration of surgery was 53 min (30–134) with 92 min (41–134) in the first and 39 min (30–117) in the second period (*p* = 0.003).

**Table 2:** Individual characteristics of conversional cases

No	Year	Type of index surgery	Type of internal hernia surgery	Case description
1	2015	RYGB	Emergency	Conversion because of no mobility and fragility with transmural lesion in biliopancreatic limb
2	2015	RYGB	Emergency	No mobility of the common channel, large amount of fluid,
3	2015	RYGB	Emergency	Internal hernia due to adhesion to colon sigmoideum, with co-existent IH; no mobility of herniated intestina and serosal lesion; relaparotomy on POD 3 with jejunal perforation, resection of segment
4	2015	RYGB	Elective/primary open surgery	Due to preknown adhesions primary open surgery
5	2015	RYGB	Emergency	Conversion because of no mobility of herniated intestine
6	2016	RYGB	Emergency	Conversion because of no mobility of herniated complete common channel; complete ileus
7	2016	RYGB	Emergency	Conversion because of hemorrhagic congestion, no mobility of intestine, lesion already at first attempt of mobilization
8	2016	RYGB	Emergency	Conversion because of lesion near jejuno-jejunostomy; adhesions, poor visibility
9	2017	RYGB	Emergency	Conversion because of fragile tissue, ileus, many adhesions after multiple surgeries
10	2017	RYGB	Emergency	Conversion because of complete ileus, infarciated jejenal segment, no laparoscopic visibility
11	2019	RYGB	Emergency	Conversion because of complete ileus, insufficient laparoscopic visibility, strangulation of jejeunum

Complications (Clavien-Dindo I–IIIb) occurred in five patients (13.5%), all of whom were operated during the first time-span (26.4% vs. 0%, *p* = 0.01) Complications ranked higher than Clavien Dindo IIIb did not occur.

Overall length of stay (LOS) was 5.3 days (1–35) with 7.7 days (2–35) in the first and 2.8 days (1–12) in the second time span (*p* = 0.019; Table 1).

## Discussion

While the occurrence of an internal hernia after gastric bypass surgery can be reduced by sufficient closure of mesenterial defects, their overall occurrence remains high [6]. In our patient population, an internal hernia was diagnosed after substantial weight loss (17.2 kg/m^2^) on average 34 months after the index procedure, which is in line with previous studies [7].

In this study, we could demonstrate that surgical therapy of an internal hernia after gastric bypass can be significantly improved by implementing institutional and surgical standards. Importantly, baseline characteristics in terms of age, sex, BMI, achieved total weight loss%, and time interval to index surgery were comparable between the two groups.

The laparoscopic approach has clear advantages, as most of the operations requiring revision occurred as a result of wound infections/complications after open surgery. After the implementation of institutional and perioperative standards in 2017, we were able to significantly reduce the conversion rate to the open approach. Accordingly, the operation time, the occurrence of complications and the length-of-stay could also be reduced.

Following our proposed standards, atraumatic handling of the vulnerable small intestine was possible by starting at the terminal ileum. From this anatomical landmark, the entire common channel up to the jejunojejunostomy was easily reposed without applying traction in the “wrong direction” and damaging the small intestine.

After this step, the alimentary limb could easily be identified starting from the pouch. This ensured a safe and atraumatic reposition of the entire small intestine before the mesenteric gaps were identified and closed.

The preparation for changing the position of the patient may seem demanding, but enables in our experience optimal exposure at all times. The additional trocars allow the use of two holding instruments which sufficiently expose the mesenteric gaps and facilitate their closure (Fig. 2). Based on previous evidence, we prefer the use of non-absorbable suture material [15]. Even if closure with absorbable suture material might be sufficient [16], we still recommend non-absorbable suture material.

If the right lower quadrant cannot be explored (e.g., because of adhesions deriving from former surgeries) the alimentary limb starting at the pouch can be followed until the jejuno-jejunostomy is reached. Before closing the Petersen’s space, it has to be assured that the biliopancreatic channel is correctly placed at left side of the mesenteric defect and the remaining small intestine at the right. Thereafter, the mesenteric defect of the jejunojejunostomy has to be explored. If the intestine cannot be easily replaced laparoscopically or if the anatomy remains unclear, we recommend converting to open surgery especially, when an ileus is present, to prevent small bowel lesions.

Generally, if a patient with suspected internal hernia is stable and without signs of peritonitis, she/he should be admitted to a certified bariatric center if timely possible.

Some limitations should be mentioned. The implementation of the targeted surgical approach was accompanied by simultaneous changes of key institutional and perioperative standards. Therefore, it is not possible to independently analyze the impact of the different key factors implemented. Of note, all revisional surgeries for suspected IH were performed by a certified senior bariatric surgeon as requested by regulations of the German Association of Visceral Surgery (DGAV). This alone may at least in part explain the observed improved outcome [12, 13]. In our experience, however, the targeted surgical approach was easy to follow and successfully performed by trainees who later qualified as senior bariatric surgeons during the study period.

## Conclusion

Our approach is an easy and safe way to perform laparoscopic internal hernia repair after intestinal bypass surgery. The focus is not the detection of the internal hernia itself, but the prevention of intestinal damage by gentle and easy reposition of the potentially vulnerable intestine. Our results show a significant improvement in outcome, even in an emergency setting for the vast majority of cases. The details described may provide valuable information for non-specialized surgeons to avoid pitfalls of surgical care of internal hernias and improve their outcome.

### Supplementary Information

Below is the link to the electronic supplementary material.

Supplementary file1 (MP4 409738 KB)

## Data Availability

The raw data needed to reproduce the above results are available. The corresponding author will make them available upon request.
